# Drusen and Pro-inflammatory Mediators in the Post-Mortem Human Eye

**DOI:** 10.4172/2155-9570.1000208

**Published:** 2012-01-27

**Authors:** Kailun Jiang, Eleanor To, Jing Z. Cui, Sijia Cao, Jiangyuan Gao, Joanne A. Matsubara

**Affiliations:** 1 Faculty of Medicine, University of Manitoba, Winnipeg, Canada; 2 Department of Ophthalmology and Visual Sciences, University of British Columbia, Vancouver BC, Canada

## Introduction

Despite significant advances in treatment, age-related macular degeneration (AMD) remains the leading cause of blindness in the elderly of developed nations [1]. Retinal cells experience functional decline with age as a result of metabolic changes and cumulative oxidative stress [2]. Early AMD is characterized by retinal pigment epithelium (RPE) dysfunction [3]. In the early stages of disease, immunomodulatory proteins and inflammatory cells accumulate in the outer retina, with an associated increase in membrane attack complex (MAC) at the RPE and choroidal interface at Bruch’s Membrane [4,5]. In some cases, accumulation of lipo-glyco-proteinacous deposits in the basal lamina, called drusen, become progressively larger and more abundant and may further cause RPE atrophy [6,7].

Growing evidence suggests a key role of inflammatory pathways in pathogenesis of AMD, triggered either by an aberrant innate immunity or autoimmunity mechanism. Variants of genes in the complement system, such as complement factor B (CFB), factor I (CFI), factor H (CFH), complement component 2 (C2), component 3 (C3) and component 7 (C7), correlate with either increased or decreased susceptibility to AMD [8,9]. In the AMD retina, immune cells such as macrophages, microglia, and giant cells localize to areas surrounding drusen deposits [10–12]. Activated microglia is capable of inducing direct damage to photoreceptors as shown by *in vitro* studies [12,13]. Proteomic studies revealed that drusen contain a number of inflammatory molecules, including C3, C5, C5b-9 and MAC [9,14]. Additionally, amyloid-beta (Aβ) and advanced glycation end products (AGE) are present [15]. In an earlier microarray study, we found that human RPE cells treated with Aβ produced strong upregulation of interleukin-1-beta (IL-1β) [16]. IL-1β is an important upstream mediator of inflammatory response and is involved in signaling macrophage activation and production of cytokines. IL-1β is capable of inducing reactive oxygen species(ROS) in RPE cells [17]. ROS trigger the release of IL-8, which recruits pro-inflammatory cells such as macrophages [16–18]. Macrophages are present in drusen deposits of AMD eyes and play a key role in promoting neovascular proliferation [19,20]. Recent studies demonstrated the presence of anti-retinal auto-antibodies in early AMD [14]. In these patients, components of drusen such as AGE may be involved in triggering autoimmune responses. *In vitro*, AGE strongly stimulates chemokine CXC motif ligand 10 and 11 (CXCL-10 and CXCL-11) production in RPE cells (unpublished observation). CXCL-11 recruits activated-T_h_-lymphocytes and B-cells and may serve as a beacon for B-cell accumulation, activation, and proliferation [21,22]. It is likely that this pathway, along with other similar ones, results in increasing tissue exposure to auto-antibodies. Altogether, our *in vitro* studies indicate that the components of drusen may be involved in the induction of inflammatory pathways facilitating AMD pathology (unpublished observation) [16]. In particular, IL-1β, CFI, radical S-adenosyl methionine domain containing 2 (RSAD2), and CXCL-10/11 were significantly upregulated. Our data also suggested activation of the JAK/STAT pathway, involved in the interferon response. STAT3, a member of the JAK/STAT pathway, was found to accumulate in the RPE cells of choroidalneovascular membranes of AMD patients [23]. With age, accumulations of drusen may increase the susceptibility of the retina to damage inflicted by these pathways. Here, we sought to verify the upregulation of the aforementioned inflammation-related gene products in the normal human retina, and to test whether changes in expression of these genes are related to the two major risk factors of AMD, age and presence of drusen.

## Methods

### Eye samples

Human donor eyes were obtained from the Eye Bank of British Columbia, Canada. Methods for securing human tissue were in compliance with the Declaration of Helsinki. The protocol was approved by the Clinical Research Ethics Board (CREB) at the University of British Columbia. All tissue samples included in this study were considered normal and excluded tissues from donors with any of the following: evidence of systemic or local infection; progressive central nervous system disease or systemic disease of unknown etiology; lymphoproliferative or myeloproliferative disorders; intrinsic eye disease or previous ocular surgery. All tissues were fixed within 20 hours of death (median time 14 hours). Eyes were divided into two age groups, a “younger” group (n=17), less or equal to 57 years and an “older” group (n=17), greater or equal to 70 years (Table 1).

### Tissue preparation and immunohistochemistry

Eye tissues were prepared as previously described by Seth et al. [4] and embedded in paraffin to obtain 6 μm sections through the pupil and optic nerve axis. Sections that included the macular region were selected for this study.

Antibodies against IL-1β, CFI, RSAD2, phosphorylated signal transducer and activator of transcription 3 (p-STAT3), CXCL-10, and CXCL-11 were used to stain sections from both age groups. Antibody dilution and source are documented in Table 2. Paraffin sections were deparaffinized and rehydrated by standard procedures. Sections underwent antigen retrieval in protease K solution (20 μg/ml, pH 8.0) for 10 minutes at room temperature. Sections were then washed twice in phosphate buffered saline (PBS, pH 7.4), treated with 0.3% H_2_O_2_ for 15 minutes, and blocked for 20 minutes with 3% horse or goat serum diluted in 0.3% Triton X (TX)-100-PBS solution to minimize non-specific staining. Tissue sections were allowed to incubate in specific primary antibodies diluted in serum and PBS with 0.3% TX-100 for 24 to 48 hours at 4°C, based on different target antigens. Two methods were employed to obtain negative control sections. In one approach, sections were incubated in the absence of primary antibody. In the second method, sections were treated with an irrelevant IgG isotype at the same concentration and in place of the primary antibody incubation (Table 2). All subsequent steps were identical. Both controls resulted in very low background staining confirming the specificity of the immunohistochemical staining patterns shown here.

After incubation in the primary antibodies, sections were thoroughly washed and incubated in secondary antibodies for 30 to 60 minutes at room temperature and rinsed twice before proceeding to alkaline phosphatase (Vector Blue, Vector Laboratories) or 3-Amino-9-ethylcarbazole (AEC, Sigma Aldrich) development. For Vector Blue chromogenic reaction, sections were first treated with Avidin-Biotinylated enzyme complex-alkaline phosphatase (ABC-AP) solution for 30 minutes and then developed for 1 hour at room temperature (counterstained with Nuclear Fast Red: Crystal Mount; Biomeda). Sections developed with AEC were pre-incubated for 30 minutes with ABC before incubation with AEC for 5 minutes (counterstained with hematoxylin). Tissue sections from both age groups were developed simultaneously to ensure comparable processing.

### Statistical analysis

**Figure 1:**
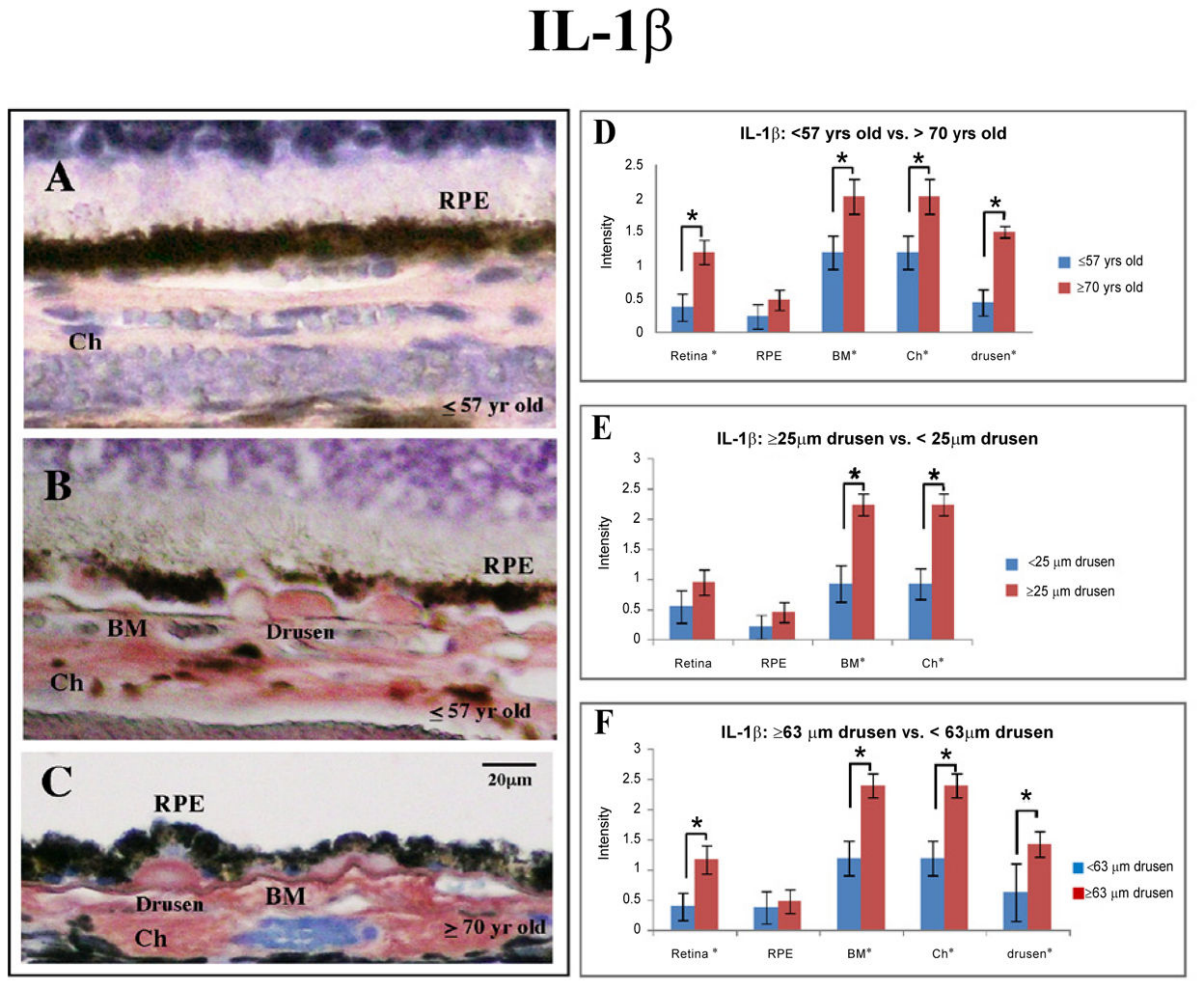
Immunostaining for IL-1β with AEC development system (red) and counterstained with Mayer’s hematoxylin (blue). IL-1β expression levels in human eye tissue, analyzed by age groups and size of drusen. (**A**) Younger group (≤ 57 yr) with drusen ≤ 25 μm; note faint immunolabeling pattern (semi-quantitative grading of 1). (**B**) Eye tissue from the younger group (≤ 57 yr) with drusen ≥ 25 μm (*arrows*). Note immunoreactivity for IL-1β in drusen and through choroid (semi-quantitative grading of 3). RPE cells are mildly immunostained. (**C**) Strong immunoreactivity in drusen, Bruch’s membrane, and choroid in eyes from older group (≥ 70 yr*,* semi-quantitative grading of 3) with drusen ≥ 63 μm (*arrows*). Scale bar 20 μm. (**D**) Aside from the retinal pigmented epithelial (RPE) cells, tissue from older group (n = 12) are more immunoreactive for IL-1β (retinal layers through to the choroid) than younger group (n = 15) (*black asterisk*, p ≤ 0.05). (**E**) Eyes with clinically significant drusen (≥ 25 μm, n = 15) were more immunoreactive for IL-1β in Bruch’s membrane and choroid compared to eyes without clinically significant drusen (n = 12). (**F**) Eyes with drusen ≥ 63 μm (n = 12) were more immunoreactive for IL-1β in the retinal layers, Bruch’s membrane, choroid, and drusen than eyes with drusen ≤ 63 μm (n = 8).

Statistical analysis was conducted by examination of four sections per eye per each antibody in a masked manner. For each molecule immunostaining intensities were scored semi-quantitatively using 40x objective lens (Eclipse 80i; Nikon, Tokyo, Japan), graded from 0 to 3. A score of 0 indicates no detectable staining above background as determined by comparison with the negative control sections. By systematic scanning through the slides, we identified ones with the strongest staining and classified them as having a score of 3. For staining levels that fall between 0 and 3, a score of 1 was given to samples with the weakest immunolabeling, while a score of 2 represented intermediate immunolabeling. We have also provided a sample range of the semi-quantitative analysis: an example of the strongest immunolabeling is shown in Figure 1, with 1A representing +1, 1B and 1C representing +3. Data were scored in the retina (ganglion cell layer (GCL), inner plexiform layer (IPL), inner nuclear layer (INL), outer plexiform layer (OPL), outer nuclear layer (ONL), inner segment (IS), and outer segment (OS)), retinal pigment epithelium (RPE), Bruch’s membrane, choroid, and drusen sites. Sub-retinal layers were scored individually; scores of all retinal layers were summed and averaged to evaluate expression level in the overall retina. Drusen size was determined using RPE cell size as reference (RPE diameter measured parallel to Bruch’s membrane was estimated at 12–14 μm) [24]. Statistical analysis was done using the Mann-Whitney test (GraphPad Software, Inc., La Jolla, CA). A one-tailed test was selected based on the directional hypothesis that accumulation of pro-inflammatory modulators increased with age and presence of drusen. Significance level was set at P ≤ 0.05.

### Categorization of drusen by size

Drusen were grouped into three categories based on their size. Tissues were initially classified to contain “observable drusen” if there are discrete lipo-proteinaceous deposits between the basal lamina of the RPE and Bruch’s membrane with diameters ≥ 4 μm. Next, we classified “clinically significant drusen” to be those with a diameter of ≥ 25 μm [24]. This is based on the fact that, during clinical exams, the limit of resolution through fundoscopy is between 25 to 30 μm. Lastly, due to the physiological significance associated with size we further sub-categorized samples into those with drusen ≥ 63 μm in diameter [24,25]. We randomly sampled 20 sections at 20x magnification from each eye to determine the presence and size of drusen in individual eyes. These categories are shown for each eye on Table 1.

## Result

### Younger eyes (≥ 57 years) versus older eyes (≥ 70 years)

In order to determine the age-related difference in expression for components of the inflammatory pathways, we compared immunoreactivity in younger eyes (≤ 57 years) with older eyes (≥ 70 years) (Table 3A). Four of the six antigens studied here demonstrated age-related changes. IL-1β immunoreactivity was significantly stronger in the retina, Bruch’s membrane, choroid and drusen deposits in tissue obtained from older group (Figure 1C, D).

**Figure 2:**
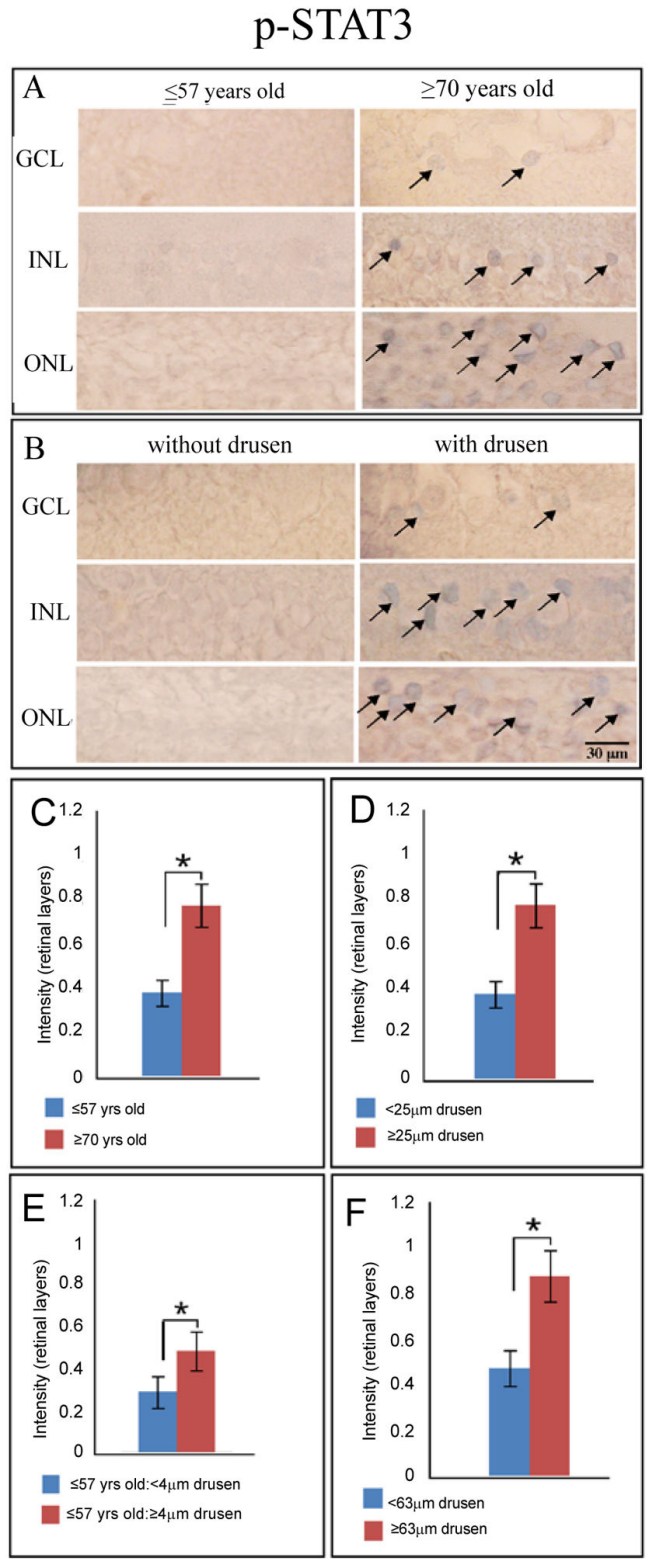
p-STAT3 levels in human eye tissue visualized with Vector Blue (blue) and counterstained with neutral red (pink). (**A**) Cells (*arrows*) in the ganglion cell layer (GCL), inner nuclear layer (INL), and outer nuclear layer are found to be immunoreactive (vector blue) for phosphorylated (activated) STAT3 in the older group (≥ 70 yr). (**B**) Similarly, immunolabeling profile is seen in eyes with drusen ≥ 25 μm regardless of age. Scale bar 30 μm. p-STAT3 accumulation significantly differs (p ≤ 0.05, *black asterisk*) in the retinal layers between the analyzed groups. STAT3 activation is greater in older eyes (**C**, ≥ 70 yr, n = 14), in eyes with clinically significant drusen (**D**, ≥ 25 μm, n = 14) and particularly in eyes with large drusen (**F**, ≥ 63 μm, n = 10). In the younger group (**E**, ≤ 57 yr, n = 14), the presence of drusen ≥ 4 μm (n = 7) is associated with increased STAT3 activation compared to eyes without drusen (n = 7).

Immunoreactivity for p-STAT3, the activated form of STAT3, was significantly stronger in the older group for the combined retinal layers (Figure 2A, C). Specifically, this observation was found to be statistically significant in the GCL, INL, and ONL. For CXCL-11, the overall retinal layers immunostained significantly stronger in the combined retinal layers, particularly OPL of younger eyes. In addition, younger eyes also showed significantly stronger CXCL-11 expression in Bruch’s membrane. Similar to CXCL-11, CXCL-10 immunoreactivity was significantly stronger in the combined retinal layers of younger eyes, specifically in the GCL. There were no discernable differences in immunoreactivity between the two age groups for CFI and RSAD2.

### Eyes with drusen: ≤25μm versus ≥25 μm (independent of age)

Next, we investigated whether the presence of clinically significant drusen (≥ 25 μm) was correlated with changes in expression levels of components of the inflammatory pathway (Table 3B). Antibodies against IL-1β localized strongly to Bruch’s membrane and choroid of eyes with drusen ≥ 25 μm. We did not observe differences in accumulation of IL-1β in the retinal layers or the RPE between the two analyzed groups (Figure 1B, C, E).

p-STAT3 stained more robustly in the combined retinal layers of drusen-containing eyes in particular, significantly higher activation of STAT3 was found in the ganglion cell layer, inner and outer nuclear layers of eyes with clinically significant drusen (Figure 2B, D). CXCL-10 demonstrated similar staining patterns to p-STAT3 in retina.

**Figure 3:**
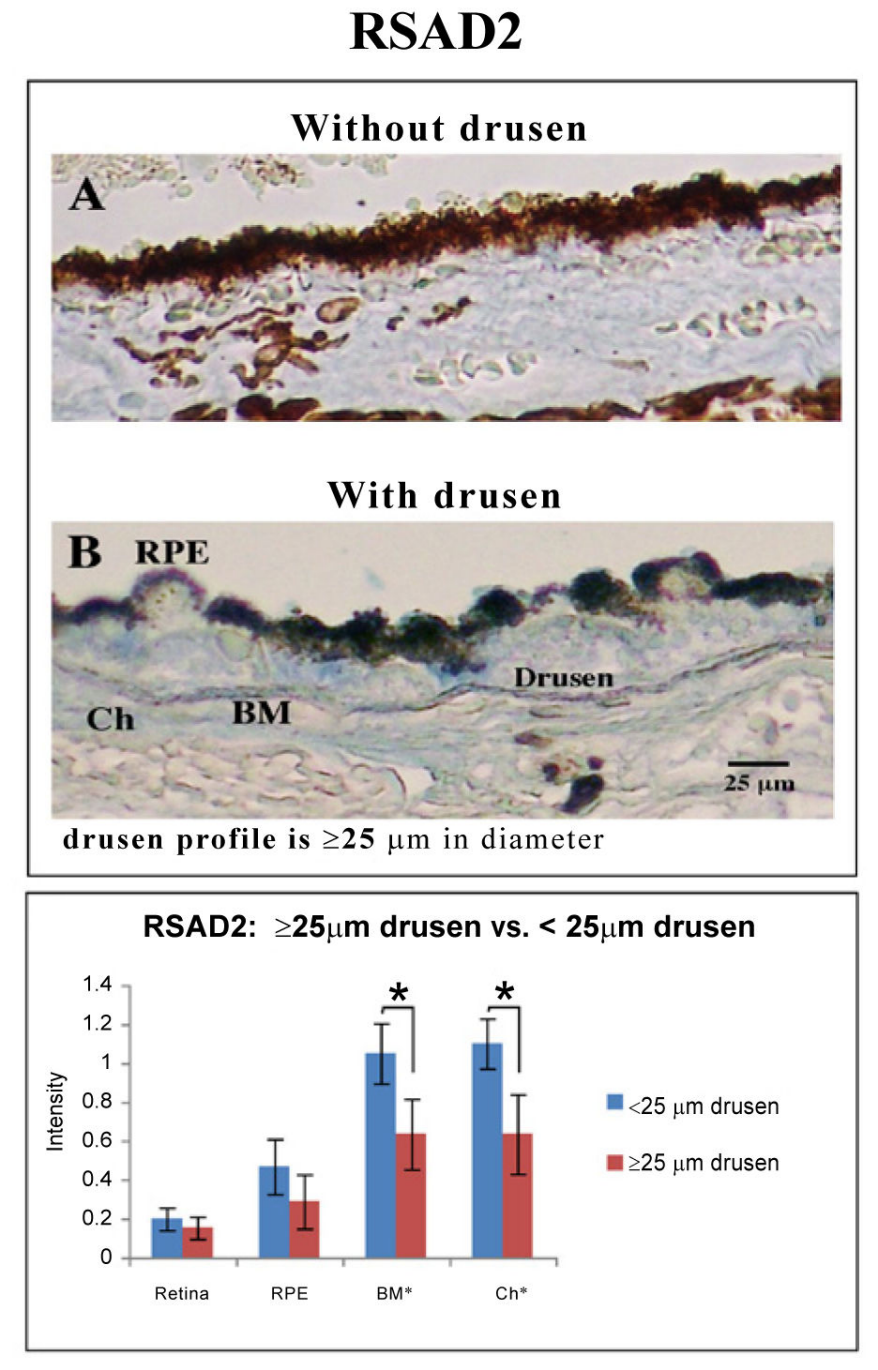
Immunostaining for RSAD2 with Vector Blue (blue) and counterstained with neutral red (pink). (**B**) Representative sections from eyes with drusen ≥ 25 μm shown that areas around drusen (≥ 25 μm), in RPE and Bruch’s membrane are more immunoreactive for RSAD2 than (**A**) the background reactivity seen in tissues with small (≤ 25 μm) or no drusen. Scale bar 25 μm. (**C**) Eyes with clinically significant drusen (≥ 25 μm, n = 15) are found to have stronger immunostaining for RSAD2 particularly in the Bruch’s membrane and choroid (p ≤ 0.05, *black asterisk*) than those without clinically significant drusen (n=13).

RSAD2, similar to IL-1β was most immunoreactive in Bruch’s membrane and through the choroid (Figure 3B, C). In contrast, CFI and CXCL-11 levels did not differ with the presence of clinically significant drusen, in this age independent analysis.

### Younger eyes (≤ 57 years): ≤ 4 μm drusen versus ≥ 4 μm drusen (controlled for age)

Individual components of drusen (Aβ, AGE) have been found to upregulate pro-inflammatory factors in cultured RPE cells (unpublished observation) [16]. Thus, it is important to evaluate for any correlation between presence of drusen and changes in the expression and/or accumulation of inflammatory factors in the eye. Although drusen ≤ 25 μm are not considered clinically significant, the pro-inflammatory Aβ is present in these deposits [26]. Thus, we compared candidate gene expression levels in younger eyes with (n=10) or without (n=7) drusen (≥ 4 μm).

**Figure 4:**
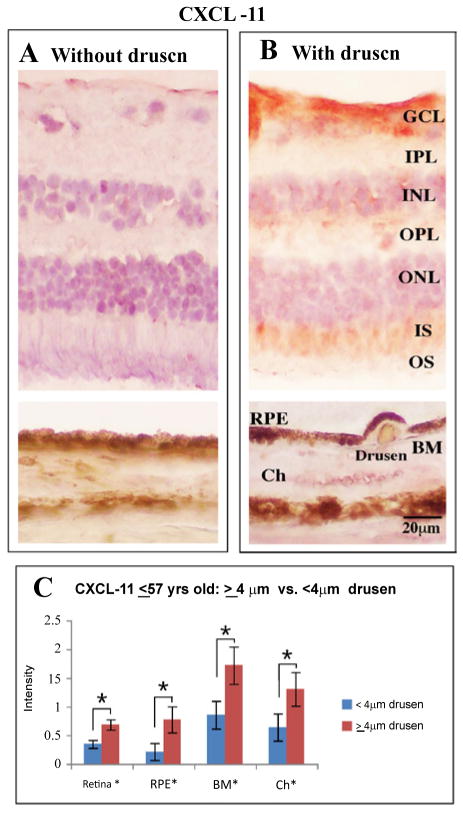
Immunolabeling for CXCL-11 with Vector Blue (blue) and counterstained with neutral red (pink). Representative sections from younger eyes (≥ 57 yr, n = 16) are shown. Samples were analyzed for the presence of drusen (≥ 4 μm, n = 9). Within the younger age-group, eyes with drusen (**B**) have greater accumulation of CXCL-11 throughout the retina and choroid. Scale bar 20μm. (**C**) Within tissue of younger eyes (≤ 57 yr, n = 16), those with any observable drusen (≥ 4 μm, n = 9) were found to stain stronger (p ≤ 0.05, *black asterisk*) for chemokine, CXCL-11 from the retina through to the choroid.

**Figure 5:**
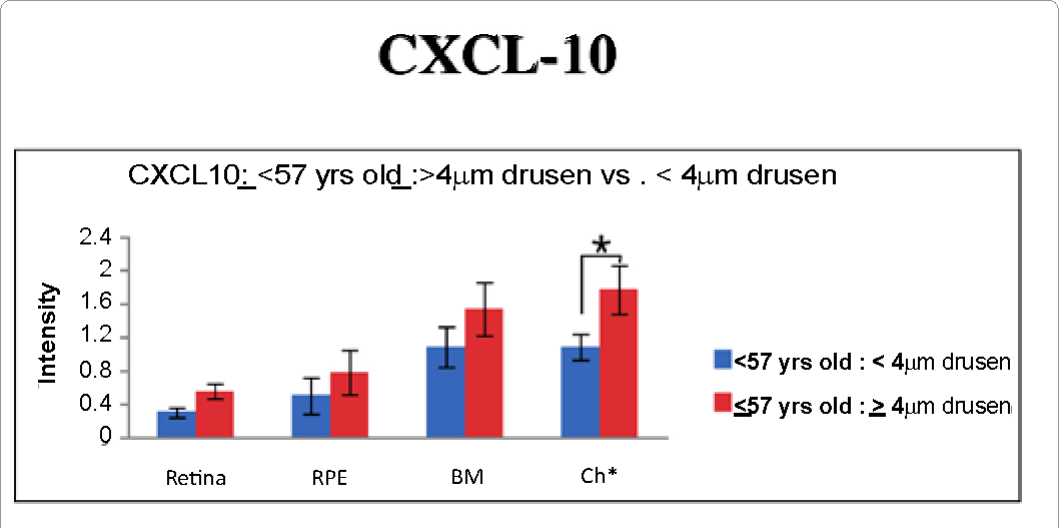
In the younger age group (≤ 57 yr, n = 15), immunoreactivity against CXCL-10 is significantly greater in the choroid of eyes with drusen (≥ 4 μm, p ≤ 0.05, n = 9).

Expression of CXCL-11was observed to be stronger in the drusen group for the combined retinal layers, RPE, Bruch’s membrane, and choroid (Table 3C, Figure 4). Specifically, in the retina, this observation was found to be statistically significant for the ganglion cell layer and outer segments. p-STAT3 was also strong in the retinal layers in young eyes with drusen, while the immunoreactivity against CXCL-10 was strong in the choroid layer of young eyes with drusen (Figure 2, 5). We found no significant difference in IL-1β and CFI immunostaining between the retinas of younger eyes with or without drusen.

### Eyes with drusen: ≤ 63 μm versus ≥ 63 μm (independent of age)

Next, we examined whether any sub-populations exist within eyes carrying drusen. This analysis was motivated by the observation that 95.5% to 98.8% of the human population has one or more drusen [27–30]. While the most frequently observed drusen are ≤ 63μm and considered relatively benign, the prevalence of drusen ≥63 μm increases with age and is considered a risk factor for age related maculopathy [24]. Thus, it is important to discern between these two sizes of drusen. The drusen diameters found in this study range from 4 μm to 126 μm. We observed that drusen ≥ 63 μm are found mostly outside of the macular area at peripheral location, while drusen ≤ 63 μm are distributed more evenly amongst foveal, parafoveal and peripheral regions (data not shown). Using the morphological classification provided by Rudulf et al. [31], drusen ≥ 63 μm identified in our study were predominantly hard drusen.

Of the 25 eyes found with drusen deposits, 12 eyes had drusen deposits that were ≥ 63 μm. In this group of eyes (≥ 63 μm), expression of IL-1β was found to be significantly stronger in the combined retinal layers, Bruch’s membrane, choroid, and drusen. Immunoreactivity to CXCL-11 was significantly stronger in the combined retinal layers of eyes with drusen ≥ 63 μm, although significant differences were not observed in individual neuronal layers of the retina (Table 3D). Interestingly, p-STAT3 immunoreactivity was stronger in Bruch’s membrane in eyes with drusen ≤ 63 μm. However, expression of p-STAT3 in combined retinal layers was stronger in eyes with drusen ≥ 63μm. In particular, the ganglion cell and the inner nuclear layers show greater p-STAT3 expression in eyes with drusen ≥ 63 μm (Table 3D). No differences in CFI, RSAD2, and CXCL-10 expression levels were observed between the two groups (Table 3D).

## Discussion

Here, we examined the expression levels of multiple candidate biomarkers of AMD by immunohistochemistry in human postmortem eye tissues and correlated the results with age and presence of drusen, two known risk factors for AMD development and progression. Most of the targets we tested were positively correlated with age and/or presence of drusen. This would be expected if drusen indeed induces inflammation via Aβ and/or AGEs [16].

### IL-1β

IL-1β is strongly implicated in the pathogenesis of chronic inflammatory diseases [32]. First, aberrant auto-upregulation of IL-1β leads to excessive inflammation. Second, it promotes angiogenesis through upregulation of VEGF, the target of choice for slowing the exudative or wet form of AMD [33]. In this study, eyes from the older group demonstrated significantly stronger immunoreactivity for IL-1β than those from the younger group. Since the tissue samples were from individuals who had no known or diagnosed eye diseases, this result suggests that older eyes are more prone to an inflamed state. This is also consistent with studies identifying age as one of the biggest risk factors of AMD [1].

Additional examination of IL-1β accumulation showed that regardless of age, eyes with clinically significant drusen have significantly stronger immunereactivity localized to Bruch’s membrane and choroid layers (Figure 1). IL-1β is secreted by a number of cell types into local environment and systemic circulation. Serum levels of IL-1β do not change significantly with aging [34]. Also, *in vitro* stimulation of RPE cells with Aβ resulted in increased IL-1β expression [16,35]. While we cannot rule out an abnormal retinal accumulation of IL-1β derived from systemic circulation, at least part of the IL-1β accumulation with aging and drusen may be explained by increased IL-1β secretion by RPE cells. Furthermore, a sub-analysis of all eyes with drusen indicated that staining for IL-1β was significantly stronger in eyes with large drusen (≥ 63 μm), which we found to be enriched in the peripheral. Peripheral drusen, while producing little structural disruption, may have indirect effects on the cells in the macular region [36]. Interestingly, peripheral drusen ≥ 63 μm is strongly associated with CFHY402H polymorphism [37]. Taken together, these results suggest that, while expression and accumulation of IL-1β in eye tissue is correlated with the presence of drusen deposits, it is drusen ≥ 63 μm that are associated with increased levels of IL-1β. It is important to note that advanced age can potentially confound our conclusions in this analysis because the prevalence of drusen ≥ 63 μm is correlated with increasing age. Thus, further studies designed to control for this confounding factor are warranted.

### p-STAT3

STAT3 is an oncogene and can be activated by IFNs; it induces transcriptional responses that block apoptosis and promote cell survival during inflammation [38,39]. In tumour cell lines, STAT3 can induce VEGF production either directly via activation of VEGF transcription or indirectly viahypoxia-inducible factor 1α [40]. We observed increased immunoreactivity for p-STAT3 with increasing age and with presence of drusen. The increase in pSTAT3 level is particularly prominent in retina with drusen ≥ 63 μm in diameter (Figure 2). In an age-controlled analysis, we continued to see STAT3 activation to be associated with presence of drusen ≥ 4 μm. These associations raise the possibility that components of drusen promote the activation of STAT3, which acts to protect the retina from apoptotic damages, and to promote exudative AMD through VEGF upregulation.

### RSAD2

Increased RSAD2 expression has been reported in age-related diseases such as atherosclerosis, the pathology of which is associated with deposition of drusen components such as Aβ and AGE [26,41]. In atherosclerosis, local inflammation could cause increased RSAD2 expression, leading to abnormal lipid accumulation [42]. Lipid accumulation in Bruch’s membrane is associated with upregulation of VEGF in the choriocapillaris of mice [43]. Previously, we found that stimulation of RPE cells with either Aβ or AGE increased RSAD2 expression [16] (unpublished observation). Here, we reported increased RSAD2 immunoreactivity in eyes with drusen, but not in aged eyes. These data indicate that drusen component such as Aβ/AGE might upregulate RSAD2 expression through induction of local inflammation. The consequent lipid accumulation may further lead to VEGF upregulation in the choriocapillaris, a result that is implicated in neovascular AMD.

### CXCL-10 and CXCL-11

RSAD2 is an antiviral protein, activated by IFN α, β, or γ [44]. Upregulation of RSAD2 suggests a role for interferon in AMD pathogenesis. We further examined this system by looking at the downstream targets of IFN-γ: CXCL-10 and CXCL-11 [21]. Both ligands are agonist of CXC receptor 3 (CXCR3). They promote chemotaxis of T-cells, B-cells, and natural killer cells [21]. Through CXCR3-mediated mechanism, CXCL-10 and 11 inhibit angiogenesis and inhibit endothelial cell proliferation (49, 50 of serum paper). Serum concentration of CXCL-10 increased dramatically with age and with the development of AMD [45,46]. Mo et al. [46] confirmed the accumulation of CXCL-10 in human eye tissue with AMD and proposed that CXCL-10 may function as a biomarker for AMD. Although we found the opposite in terms of age related changes in CXCL-10 concentration, this difference may be accounted for by the different methods used: serum versus histology. However, CXCL-10 accumulation was determined to be greater in eyes with clinically significant drusen. This trend continues in the younger age group.

Not much is known about the effects of aging on CXCL-11 levels. CXCL-11 immunohistochemical level is higher in younger eyes and no difference in staining intensity exists between those with and without clinically significant drusen (Table 3). However, CXCL-11 levels were higher in those eyes with drusen ≥ 63 μm. Moreover, CXCL-11 level was higher in tissue of the younger group with drusen (≥ 4 μm). These findings suggest that the relationship between drusen and CXCL-11 is complex. With normal aging, the macrophage population shifts from the pro-inflammatory M1 to the anti-inflammatory M2, which are associated with diminishing CXCL-11 expression [47]. Cytokines such as CXCL-11 and IL-1β are markers for M1 macrophage phenotype. CXCL-11 is also upregulated in AMD [47]. Our data suggest that CXCL-11 is an early precursor of disease progression, upregulated in younger eyes with drusen. This may warrant further investigation to determine its future role as an early indicator of AMD disease progression.

## Summary

**Figure 6:**
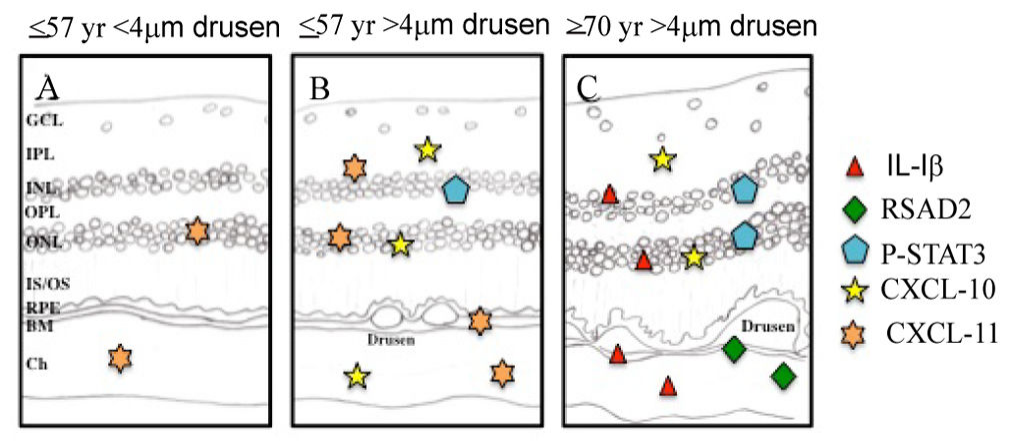
Summary diagram depicting the distribution of IL-1β, RSAD2, p-STAT3, and CXCL-10/11 immunoreactivity in post-mortem eye tissue with respect to aging and present of drusen. Icons denote the relative immunostaining of each molecule in a given retinallocation.

With age and drusen accumulation, the environment of the eye tends to shift more towards a pro-inflammatory state (Figure 6) through either IL-1β and/or possibly IFN pathways. Little change is induced in the complement inhibitor, CFI, in association with drusen, suggesting a failure to activate protective mechanisms against complement activation. Alternatively, the lack of change in CFI accumulation level could indicate that that the pro-inflammatory actions of drusen may not yet be enough to trigger inhibition of the complement cascade in healthy eye tissue. Here, we identified three inflammatory molecules in the postmortem human eye, p-STAT3, CXCL-10, and CXCL-11, to be strongly correlated with the presence of drusen within a population controlled for age (≤57 years old, Figure 6). This pattern of accumulation suggests that these molecules are closely involved with the presence of drusen rather than with aging, and may represent a predisposition toward AMD pathogenesis. A recent microarray study on mouse RPE cells reported expression changes in over 315 genes associated with advanced age [48]. Many pathways they identified were also found in our prior microarray study (unpublished observation) [16]. *Similarly, Chen et al.* [49] *found that the mouse neuroretina displayed age-related upregulation of the complement cascade and activation of retinal microglia* [49]. *Curiously, neither study found* expression changes in any of the gene products examined in this study. It is important to note that drusen-like deposits have not been documented in wild-type-mouse eyes [50]. Thus, one interpretation for this discrepancy may be that the genes identified in our microarray studies, and specifically the gene products studied here reflect a drusen-specific response rather than a general response to aging. Our studies now set the stage for future experiments testing the function of each of these potential mediators in the pathogenesis of AMD. One approach might involve assessing the response of each mediator following Aβ and/or AGE injection into young wild-type mice to determine degree of mimicry to AMD pathology.

## Figures and Tables

**Table 1:** Donor List.

Donor	Age	Sex	Diagnosis	IL-1β	CFI	STAT3	CXCL-11	CXCL-10	RSAD2	No Drusen	Drusen <25μm	Drusen ≥25μm	Drusen ≥63μm
**≥ 70 yr Old**													
1	74	M	sub-arachnoid hemorrhage	X	X		X	X		X			
2	70	F	glioblastoma multiform	X	X	X	X	X	X			X	X
3	71	F	lung cancer	X	X	X	X	X	X			X	X
4	70	M	hepatitis B and colon cancer with metastasis		X	X	X	X	X		X		
5	74	M	bowel cancer	X	X	X			X			X	
6	75	M	heart disease (cardiac arrest)	X	X	X	X	X	X			X	X
7	72	M	pancreatic cancer	X	X	X	X	X	X			X	X
8	71	M	colon cancer with metastasis	X	X	X	X	X	X			X	X
9	74	M	bone cancer with metastasis	X	X	X	X	X	X			X	X
10	72	F	gastroenteritis and chronic renal failure	X	X	X	X	X	X			X	X
11	72	M	respiratory arrest		X	X	X	X	X			X	
12	80	M	renal failure	X	X	X	X	X				X	X
13	70	M	cardiogenic arrest	X	X	X	X	X	X			X	X
14	72	F	large left cerebral vascular accident			X			X		X		
15	80	F	not available		X		X	X				X	X
16	72	M	renal cancer	X	X	X	X	X	X			X	
17	73	M	lung and liver cancer				X	X		X			
**≤ 57 yr Old**													
18	47	F	lung cancer with mets	X	X	X	X	X	X		X		
19	46	F	lung cancer		X	X	X	X	X		X		
20	46	F	brain tumor		X	X	X	X	X			X	
21	48	F	liver cancer, cirrhosis	X	X	X	X	X	X			X	X
22	56	F	breast cancer with brain metastasis	X	X	X	X	X	X	X			
23	54	F	Glioblastoma	X	X	X	X	X	X		X		
24	55	M	Prostate cancer	X	X	X	X	X	X	X			
25	55	M	metastatic esophageal cancer		X	X	X	X			X		
26	42	F	lung cancer with metastasis	X	X	X	X		X	X			
27	17	M	multiple trauma due to car accident	X	X	X	X	X	X	X			
28	57	M	bladder cancer		X		X	X	X		X		
29	47	M	appendix cancer	X	X	X	X	X	X	X			
30	55	F	lung cancer	X		X						X	
31	55	F	lung cancer	X	X		X	X	X			X	
32	44	M	Brain cancer	X	X		X	X	X			X	X
33	28	F	ovarian cancer with metastasis	X	X	X	X	X	X	X			
34	35	F	cercical cancer with metastasis	X	X	X	X		X	X			

**Table 2:** Proteins studied and antibodies used.

Protein/Marker of Inflammation	Results of stimulation studies	Description	Primary Antibody	Irrelevant IgG	Working Concentration	Manufacturer	Detection System
IL-1 beta	Aβ	Interleukin-1 beta, produced by activated macrophage, mediates inflammatory response including cellular proliferation, differentiation, and apoptosis	Mouse monoclonal anti-human IL-1 beta	Mouse IgG1	1 to 200	Sigma Aldrich	AEC; Sigma Aldrich
CFI	Aβ	Complement factor I is a serine protease that inactivates C3b and C4b	Mouse monoclonal anti-human CFI	Mouse IgG1	1 to 250	Lifespan Biosciences	Vector Blue; Vector Laboratories
p-Stat3	AMD membranes	Stat3 is an important signalling molecule for cytokines/growth factor receptors. Here we stained for activated Stat3 (Phospho-Stat3 (Tyr705) (D3A7)). STAT3 is part of the JAK/STAT pathway.	Rabbit monoclonal anti-mouse phospho-Stat3	Rabbit IgG	1 to 100	Cell Signaling Technology	Vector Blue; Vector Laboratories
CXCL-11	AGE	IFN-inducible T cell α chemoattractant is a member of the C-X-C chemokine family and is expressed in IFN-γ-treated astrocytes, monocytes, keratinocytes, bronchial epithelial cells and neutrophils	Rabbit polyclonal anti-human CXCL-11	Rabbit IgG	1 to 100	Santa Cruz Biotechnology, INC	AEC; Sigma Aldrich
CXCL-10	Early biomarker of AMD	CXCL-10 (IP-10) is a member of the C-X-C chemokine family (pro-inflammatory)	Mouse monoclonal anti-human IP-10	Mouse IgG1	1 to 100	Santa Cruz Biotechnology, INC	AEC; Sigma Aldrich
RSAD2	Aβ, AGE	RSAD2 is involved in antiviral defence (against Hep C, cytomegalovirus, and HIV-1)	Rabbit polyclonal anti-human RSAD2	Rabbit IgG	1 to 100	Santa Cruz Biotechnology, INC	Vector Blue; Vector Laboratories

* Antibody detects STAT3 when STAT3 is phosphorylated at tyrosine705. Do not cross react with phopho-EGFR or other phosphor-tyrosine STAT proteins.

**Table 3:** Semiquantitative analysis of IL-1β, CFI, RSAD2, STAT3, and CXCL-11immunoreactivity.

		A	B	C	D
		≥70 yrs old vs ≤57 yrs Old	≥25 μm drusen vs <25 μm drusen	≤57 yrs old ≥4 μm drusen vs <4 μm drusen	≥63 μm drusen vs <63 μm drusen
IL-1β	Retina	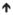 *	-	-	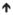 *
	RPE	-	-	-	-
	BM	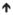 *	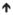 *	-	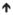 *
	Ch	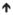 *	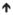 *	-	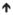 *
	Drusen	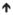 *	NA	NA	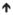 -
RSAD2	Retina	-	-	* 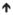	-
	RPE	-	-	-	-
	BM	-	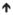 *	-	-
	Ch	-	*	-	-
	Drusen	-	NA	NA	-
p-STAT3	Retina	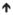 * (GCL/INL/ONL)	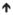 *(GCL/INL/ONL)	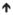 *	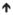 *(GCL/INL)
	RPE	-	-	-	-
	BM	-	-	-	* 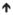
	Ch	-	-	-	-
	Drusen	-	NA	NA	-
CXCL-10	Retina	* (GCL) 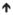	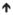 *	-	-
	RPE	-	-	-	-
	BM	-	-	-	-
	Ch	-	-	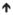 *	-
	Drusen	-	-	-	-
CXCL-11	Retina	* (OPL) 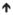	-	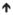 *	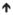 *
	RPE	-	-	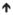 *	-
	BM	* 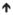	-	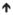 *	-
	Ch	-	-	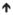 *	-
	Drusen	-	NA	NA	-
CFI	Retina	-	-	-	-
	RPE	-	-	-	-
	BM	-	-	-	-
	Ch	-	-	-	-
	Drusen	-	NA	NA	-

Mann-Whitney test (one-tail unequal variance) with significance set at p≤ 0.05 (*black asterisk*). Arrowhead denotes population with stronger immunolabeling.
